# Improving health literacy through group antenatal care: a prospective cohort study

**DOI:** 10.1186/s12884-017-1414-5

**Published:** 2017-07-14

**Authors:** Jody R. Lori, Henrietta Ofosu-Darkwah, Carol J. Boyd, Tanima Banerjee, Richard M. K. Adanu

**Affiliations:** 1 Department of Health Behavior and Biological Sciences, University of Michigan, School of Nursing, 400 N. Ingalls Bldg, Ann Arbor, MI 48109 USA; 2 Ghana Health Service, Manhyia District Hospital, Kumasi, Ghana; 3 Institute for Health Care Policy & Innovation, University of Michigan, NCAC Bldg 16, SPC 2800, Ann Arbor, MI 48109 USA; 4 School of Public Health, University of Ghana, PO Box GP4236, Accra, Ghana

## Abstract

**Background:**

To examine whether exposure to group antenatal care increased women’s health literacy by improving their ability to interpret and utilize health messages compared to women who received standard, individual antenatal care in Ghana.

**Methods:**

We used a prospective cohort design. The setting was a busy urban district hospital in Kumasi, the second most populous city in Ghana. Pregnant women (*N* = 240) presenting for their first antenatal visit between 11 and 14 weeks gestation were offered participation in the study. A 27% drop-out rate was experienced due to miscarriage, transfer or failure to return for follow-up visits, leaving 184 women in the final sample. Data were collected using an individual structured survey and medical record review. Summary statistics as well as two sample t-tests or chi-square were performed to evaluate the group effect.

**Results:**

Significant group differences were found. Women participating in group care demonstrated improved health literacy by exhibiting a greater understanding of how to operationalize health education messages. There was a significant difference between women enrolled in group antenatal care verses individual antenatal care for preventing problems before delivery, understanding when to access care, birth preparedness and complication readiness, intent to use a modern method of family planning postpartum, greater understanding of the components of breastfeeding and lactational amenorrhea for birth spacing, and intent for postpartum follow-up.

**Conclusion:**

Group antenatal care as compared to individual care offers an opportunity to increase quality of care and improve maternal and newborn outcomes. Group antenatal care holds the potential to increase healthy behaviors, promote respectful maternity care, and generate demand for services. Group ANC improves women’s health literacy on how to prevent and recognize problems, prepare for delivery, and care for their newborn.

**Electronic supplementary material:**

The online version of this article (doi:10.1186/s12884-017-1414-5) contains supplementary material, which is available to authorized users.

## Background

While significant progress was made towards meeting Millennium Development Goals 4 and 5, the quest remains to end preventable maternal and perinatal deaths. Over the past two decades, there has been a focus on increasing access to the recommended four antenatal care (ANC) visits and Basic Emergency Obstetric Care (BEmONC). Globally, this focus has been successful with more than 71% of births assisted by a skilled birth attendant in 2014, compared to 59% in 1990 and concomitantly, a decline in under-five mortality rates from 90 to 43 deaths per 1000 live births [1].

However, it is clear that more needs to be done. It is estimated that every year over 300,000 women die from complications related to pregnancy and childbirth, 2.6 million stillbirths occur, and 2.8 million newborns continue to die during their first 28 days of life [2, 3]. Antenatal care has been identified as a key strategy to maintaining a health pregnancy and promoting thriving families and communities [4]. If health literacy was improved and the use of skilled birth attendants increased further, women and newborn lives could be saved – and morbidity drastically reduced [1, 2].

Initially considered only as a patient’s ability to read and understand written information, health literacy is now more broadly defined as a person’s ability to acquire or access information, understand, and finally use the information in ways that promote and maintain good health [5, 6]. Despite a burgeoning emphasis on health literacy in high-resource countries [7], there are a dearth of studies to improve health literacy in low and middle income countries, particularly interventions focused on maternal health literacy [8]. To reduce inequalities in maternal and newborn outcomes, an approach of contextually appropriate health messaging must be constructed [9].

### Antenatal care

Focused Antenatal Care, introduced in early 2000 emphasizes quality over quantity and consists of a well-defined set of activities in a four-visit model with individuals receiving care from a consistent provider for continuity of care [10]. However, recent studies have found the reduced visit model associated with increased perinatal mortality and less satisfaction with care among pregnant women [11, 12]. Current recommendations support a shift toward coverage of ANC content rather than contact with an agreed upon set of key ANC services (i.e. full protection against tetanus toxoid, iron-folate supplementation for at least 90 days, counseling on danger signs, etc.) [13].

Alternatively, group care provides ANC content in small groups of women at similar gestational age and facilitated by a consistent provider. Women remain in the same group throughout their pregnancy, thereby enhancing information sharing and peer support among group members [14]. Widely accepted components of group care include sitting in a circle, interactive learning, planned activities, stability of group members and provider, and creating an environment where women learn from and support one another [15, 16].

Recent studies examining individual ANC in low-resource settings, have highlighted several problems with the way current care is most often delivered. Very little time is spent with pregnant women, important information is not reinforced, efficiency is poor, relationships between providers and clients are not fostered, and there is a lack of patient centeredness [17, 18]. Additionally, research has shown very low understanding by pregnant women of the actions required for birth preparedness and complication readiness [19–21]. For example, knowledge on the risks associated with vaginal bleeding, convulsions/fits, severe headaches with blurred vision, fever and weakness, severe abdominal pain, and fast or difficult breathing is often misunderstood by pregnant women [22].

A group format for delivery of care has advantages over individual care that include improved efficiency, access, patient satisfaction, provider satisfaction, and health outcomes [15, 23]. Similar to other types of group care, group ANC visits have been found to be acceptable to pregnant women and have been associated with improved outcomes in preterm birth, breast feeding initiation, patient satisfaction, and family planning in the United States using the Centering Pregnancy model [16, 24–27]. Globally, the feasibility of implementing group ANC has been examined in Iran, Tanzania, Malawi, and Ghana and found to be acceptable to both pregnant women and providers [14, 25, 28]. Group ANC holds the potential for not only increasing patient satisfaction, but also increasing a mother’s knowledge and ability to utilize the health messages she receives during antenatal care.

The aim of this study was to examine whether exposure to group ANC increases Ghanaian women’s health literacy by improving their ability to interpret and utilize health messages impacting health related behaviors and ultimately improving birth outcomes. The overall goal was to quantify the potential impact of group ANC in a low-resource setting.

## Methods

### Description of the group ANC model

The group ANC model used for this study was modified from a curriculum initially developed by the American College of Nurse-Midwives to mobilize communities in low-resource countries for early problem identification of pregnancy related problems and referral [29]. Using the WHO Standards for Maternal and Neonatal Care [30] the group ANC model was developed and tested for acceptability and feasibility for the first time in a clinical setting in Ghana [14].

A Facilitator’s Guide provides step-by-step details on how to conduct each of the 7 group ANC visits. Seven modules were developed covering the essential elements of ANC [31–35] (Table 1). The Facilitator’s Guide also includes chapters on preparing for and implementing group care, becoming a facilitator, enhancing adult learning, respectful maternity care, and monitoring for program quality, performance, and fidelity. The model uses a collaborative approach between providers and pregnant women with respect for all types of knowing.

**Table 1:** Group Antenatal Care Modules

Module	Topic
ANC Visit 1	Introduction to Group ANC and Topics
ANC Visit 2	Self- Care and Preventing Problems During Pregnancy
ANC Visit 3	Danger Signs
ANC Visit 4	Birth Preparedness and Complication Readiness
ANC Visit 5	Preventing Problems After Your Baby is Born
ANC Visit 6	Family Planning and Exclusive Breastfeeding
ANC Visit 7	Preventing and Recognizing Newborn Problems

Following the initial ANC visit, pregnant women are grouped into small groups with 12 women of similar gestational age. Prior to the start of each group, blood pressure, weight, and a urinalysis are measured on each woman. She then receives an individual assessment with the provider to measure fundal height, listen to fetal heart tones, and answer any questions she prefers not to raise in the group. Pregnant women and providers then sit in circle facing one another for a 60-min facilitated discussion. The teaching component uses strategies such as story-telling, peer support, demonstration and teach-back – capturing and sharing experiences among the pregnant women to enhance its effectiveness. Improving health literacy is incorporated as an integral part of clinical practice within the model. Evidence-based information is presented in a non-hierarchical, patient centered, participatory manner.

Because the model was developed for use in low-resource settings and with women who often have not had the opportunity to attend formal school, picture cards are used as visual images to enhance communication and learning in the group setting [14, 21]. They provide a mechanism to envision new concepts and ideas. The picture cards provide a valuable group discussion and learning aid to stimulate thinking and reflection, dialogue, and learning among participants. Content is repeated multiple times in multiple ways to enhance retention including: 1) auditory by listening to stories and signs of problems; 2) visual through use of demonstration and picture cards; 3) kinesthetically by practicing actions and “handling” picture cards; and 4) reminder pictures for home use.

### Study setting

Ghana is a low income country in sub-Saharan West Africa with a maternal mortality ratio of 319 per 100,000 [2], a perinatal mortality rate of 38 per 1000 live births, and an infant mortality rate of 41 deaths per 1000 live births [36]. While 87.3% of women in Ghana surveyed had attended the minimum standard of 4 ANC visits, 27% gave birth alone or with a non-skilled attendant [36]. Only 22.8% of newborns in Ghana received the recommended postnatal check-up within the first 2 days of life between 2012 and 2014 [36]. Since 2008, there has been only a marginal decline (3%) in neonatal mortality within Ghana [36].

### Study design

A prospective cohort design was used for this study. The comparison group received the standard individualized focused ANC by the same group of providers. The teaching component for women in individual care consisted of the midwife providing information in a lecture format to all women who presented for care that day on standard ANC educational content (i.e. danger signs, breastfeeding, birth preparedness and complication readiness, etc.) prior to their individual appointment with the midwife. The same educational content was presented as a facilitated discussion in the intervention group. Other than group vs. individual care, the two groups received identical antenatal treatment following the clinic guidelines [10]. Women enrolled in the study were encouraged to attend 7 ANC visits following the initial enrollment visit every 4 weeks until 36 weeks gestation and then every 2 weeks until 40 weeks gestation. Women were followed longitudinally from the time of entry into ANC through the postpartum period.

Institutional review board approval for the study was obtained from the University of Ghana Noguchi Memorial Institute for Medical Research; the Kwame Nkrumah University of Science and Technology Committee on Human Research, Publications and Ethics; and the University of Michigan’s Institutional Review Board.

### Sample

A facility-driven convenience sample of 240 Ghanaian women presenting for their first ANC visit between 11 and 14 weeks gestation, at a busy urban district hospital were recruited for the study. Any woman over the age of 18 years, who spoke English or Twi, was currently between 11 and 14 weeks gestation, and enrolling for ANC at the identified district hospital clinic was offered participation in the study. The research assistant met with each woman in a private area of the clinic to explain the study, answer questions, and obtain informed consent. A written informed consent was used. If women were unable to read, the research assistant read the document to them. All women provided written consent through a signature or mark entered onto the informed consent document. Every other woman presenting for her first visit, who agreed to participate in the study, was alternately enrolled into either group or individual care. Women meeting inclusion criteira were recruited consecutively until 10 groups consisting of 12 participants and 120 participants for individual care were reached. No individual refused participation.

Sample size was set by power calculations for a two group continuity corrected chi-square test with a 0.05 two-sided significance level and 80% power to detect a difference in knowledge acquisition between the two groups. The calculations were performed to detect a medium size effect of 0.5 as defined by Cohen [37]. Power analysis was conducted with nQuery Advisor 7.0 software [38]. From these calculations, a sample size of 73 was required per group. Anticipating the potential for a high attrition rate during the course of the study because of the known mobility patterns and potential loss to follow up in this peri-urban community, we employed an over sampling strategy.

### Measures

Demographic data were collected on all pregnant women at the beginning of the study. The study utilized individual survey questions and chart review for data collection. Survey questions were adapted from the Home Based Life Saving Skills evaluation toolkit [29]. The survey was assessed for both face and content validity by US and Ghanaian researchers familiar with antenatal care research in Ghana in particular, and sub-Saharan Africa in general. Questions were refined to assure comprehension of the concepts in the Ghanaian context. The survey included 37 questions to capture the knowledge gained by pregnant women during their experiences with antenatal care; 4 short answer, 18 dichotomous (yes/no) questions, and 15 recall questions to assess self-care knowledge, birth preparedness, complication readiness, breastfeeding knowledge, and postpartum danger signs. Measurement tools are available, Additional files 1, 2, and 3.

### Study data collection

The individual survey took approximately 20–30 min to complete. Women were informed they could refuse to answer any question or stop the survey at any time. Data were collected by the research assistant following birth using a face-to-face individual structured survey. Due to low literacy, the survey was administered verbally by the research assistant. Women were given an incentive of baby items worth approximately 8 US dollars upon completion of the survey. A medical record review was also conducted to obtain information on birth weight, number of ANC visits, mode of delivery, and maternal and perinatal morbidities and mortalities.

### Data analysis

All data were first entered into an excel spreadsheet and transferred to SAS 9.4 (SAS Institute Inc., Cary, NC, USA) for analyses. Summary statistics based on mean, standard deviation, or frequency were carried out for the exploratory analysis. Two sample t-tests or chi-square tests were performed to evaluate the group effect for demographic, individual surveys, and chart view items depending on the type of variables (continuous or categorical). Significance was determined at *p* < .05.

## Results

**Fig. 1:**
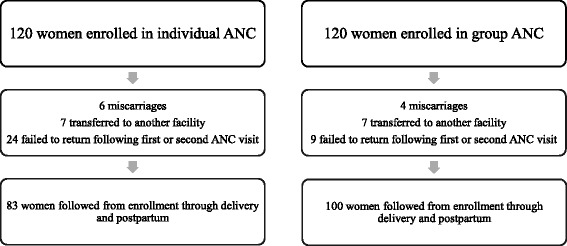
Flow Diagram of Enrollment

Two-hundred forty women were enrolled in the study. Fifty-seven women dropped from the study due to miscarriage, transfer to another facility, or failure to return after initial visit (cause unknown) for a retention rate of 72.6%. One-hundred eighty-three women were followed from the time of enrollment through postpartum. One hundred women participated in group care and 83 women received individual focused antenatal care (Fig. 1).

The average age of women was 27.9 years (SD = 5.7). There was a significant, although not clinical, difference in age between the groups with women enrolled in group care on average 2 years older than women enrolled in individual care (28.8 vs. 26.7 years). Additionally, more individuals assigned to group care identified their religious affiliation as Christians (67.3%) verses Muslim (30.6%). Results were stratified during analysis to examine for any significant differences based on demographic differences. There were no significant differences in parity, age, and religious affiliation between groups on all outcome measures. Overall, only 32.9% of all women in the study reported completing any high school or higher education. Although not statistically significant, approximately 35% of women enrolled in individual care reported they were unable to read or write vs. 25% of women enrolled in group care. See Table 2 for a full description of demographic and descriptive statistics.

**Table 2:** Demographic Data

	Group (*N* = 100)	Individual (*N* = 83)	*p* value
Age at last birthday			
Mean (SD)	28.8 (5.8)	26.7 (5.4)	0.02*
Religious following			
Missing	2	2	
None	2 (2.0%)	0 (0.0%)	
Christian	66 (67.3%)	43 (53.1%)	0.04*
Muslim	30 (30.6%)	38 (46.9%)	
Marital status			
Married	73 (73.0%)	57 (68.7%)	0.52
Single	27 (27.0%)	26 (31.3%)	
Ever attended school			
Missing	1	0	
No	13 (13.1%)	13 (15.7%)	0.63
Yes	86 (86.9%)	70 (84.3%)	
Highest level of school attended			
Missing	13	15	
Primary	21 (24.1%)	13 (19.1%)	
Jr. High school	36 (41.4%)	34 (50.0%)	0.68
Sr. High school	18 (20.7%)	11 (16.2%)	
Tertiary	12 (13.8%)	10 (14.7%)	
Able to read			
Missing	1	0	
No	25 (25.3%)	29 (34.9%)	0.15
Yes	74 (74.7%)	54 (65.1%)	
Able to write			
Missing	0	1	
No	24 (24.0%)	29 (35.4%)	0.09
Yes	76 (76.0%)	53 (64.6%)	
Number of pregnancies			
Mean (SD)	3.2 (1.7)	2.9 (1.5)	
Range	(1.0–7.0)	(1.0–7.0)	0.20
Primigravida	17 (17%)	15 (18%)	
Number of living children			
Mean (SD)	1.6 (1.5)	1.3 (1.3)	0.22
Range	(0.0–5.0)	(0.0–5.0)	
Travel time to district hospital			
Mean (SD)	1.1 (0.3)	1.1 (0.3)	0.9
Range	(1.0–3.0)	(1.0–2.0)	
Type of transportation to hospital			
Missing	7	6	
Car	5 (5.4%)	2 (2.6%)	
Walk	14 (15.1%)	16 (20.8%)	0.46
Taxi	17 (18.3%)	18 (23.4%)	
Tro-tro (mass transit)	57 (61.3%)	41 (53.2%)	

*p ≤ .05

### Improved self-care knowledge to prevent problems

Following delivery, women were asked to recall what they learned during ANC to help them care for themselves during pregnancy. Women in group care recalled learning more self-care behaviors to help them remain healthy during their pregnancy such as, drinking plenty of fluids (p = <0.01) and getting additional rest (*p* = 0.01). Twice as many women enrolled in group care (38%) reported they were instructed to watch for problems and report to the clinic when they identified a problem than women receiving individual care (18.3%) *p* = 0.01. Women in group care also reported they were more often instructed by their midwife on nutrition and to eat adequate foods. Overall, there was a significant difference in the overall number of self-care measures recalled among women attending group ANC (M = 6.0, SD = 0.87) vs. individual ANC (M = 5.05, SD = 1.21), (*p* = 0.01) (Table 3).

**Table 3:** Self-Care Knowledge to Prevent Problems

Topic	Group (*n* = 100)	Individual (*n* = 83)	*p*-value
Midwife talked about nutrition	100 (100%)	78 (94%)	0.01**
Midwife talked about warning signs	100 (100%)	82 (98.8%)	0.27
Instructed to rest to prevent problems	62 (62%)	31 (37.3%)	0. 01**
Instructed to sleep under a mosquito net	91 (91%)	78 (94%)	0.45
Instructed to eat an extra meal daily	97 (97%)	72 (86.7%)	0.01**
Instructed to drink plenty of fluids	96 (96%)	60 (72.3%)	<0. 01**
Instructed to watch for problems & report to clinic	38 (38%)	15 (18.3%)	0.01**
Instructed to take iron tablets to prevent problems	96 (96%)	75 (90.4%)	0.13

*p ≤ .05; **p ≤ .01

### Birth preparedness and complication readiness

Women enrolled in group ANC visits were more likely to have arrangements made in advance for emergency transport should a problem occur (*p* = 0.001) and to have saved money in preparation for birth (p = <0.001). They also reported more frequently discussing with their midwife where they would deliver (*p* = 0.001). Women participating in group ANC reported being more influenced by their midwife during ANC on their choice of where to deliver (*p* = 0.001) than women receiving individual care (Table 4). Overall, women attending group care were able to recall more danger signs (M = 3.49, SD = 0.88) compared to women in individual care (M = 3.12, SD = 0.93), (*p* = 0.01).

**Table 4:** Birth Preparedness and Complication Readiness

Topic	Group (*n* = 100)	Individual (*n* = 83)	*p*-value
Arranged emergency transport	98 (98%)	69 (83.1%)	0.01**
Arranged money to prepare for delivery	99 (99%)	67 (80.7%)	<0.01**
ANC care influenced place of delivery	62 (62%)	28 (33.7%)	0. 01**
Talked with midwife about where to deliver	51 (51.5%)	20 (24.7%)	0. 01**
Arranged layette for baby	92 (92%)	76 (91.6%)	0.92
Obtained supplies for delivery	95 (95%)	79 (95.2%)	0.96
Knew Danger Signs			
Vaginal bleeding	99 (99%)	81 (97.6%)	0.46
Fever	24 (24.0%)	15 (18.1%)	0.33
Pain in breast or abdomen	46 (46%)	24 (28.9%)	0.02*
Swollen face or hands	72 (72%)	61 (73.5%)	0.82
Headache/blurred vision	91 (91%)	64 (77.1%)	0.01**
Reduced or no fetal movement	17 (17%)	14 (16.9%)	0.98

*p ≤ .05; **p ≤ .01

### Breastfeeding knowledge

Women attending group ANC had significantly more knowledge on practices to promote exclusive breastfeeding than women receiving standard, individual care. More women enrolled in group care (90%) vs. individual care (66.3%) were aware to start breastfeeding as soon as possible after birth (p = <0.01) and a greater percentage (90% vs. 75.9%) reported they should breastfeed exclusively until the baby is at least 6 months of age (p = <0.01) (Table 5).

**Table 5:** Breastfeeding Knowledge

Topic	Group (*n* = 100)	Individual (*n* = 83)	*p*-value
Start to breastfeed as soon as possible after delivery	90 (90%)	55 (66.3%)	<0. 01**
Breastfeed every 2–4 h during day	79 (79%)	53 (63.9%)	0.02*
Breastfeed at least once a night	16 (16%)	1 (1.2%)	<0. 01**
Breastfeed until baby is at least 6 months old	90 (90%)	63 (75.9%)	<0.01**
Do not give water to your baby	94 (94%)	75 (90.4%)	0.36
Do not give supplemental feedings	84 (84%)	72 (86.7%)	0.61

*p ≤ .05; **p ≤ .01

### Newborn and postpartum self-care knowledge

More women receiving group care reported discussing newborn problems during their ANC visits with the midwife (p = <0. 01). They also were more likely to be aware that excessive bleeding postpartum is a problem (<0. 01) and a significantly greater number of women enrolled in group care intended to use postpartum family planning (p = <0. 01) (Table 6).

**Table 6:** Newborn and Postpartum Self-care Knowledge

Topic	Group (*n* = 100)	Individual (*n* = 83)	*p*-value
Knows excessive bleeding postpartum is a danger sign	94 (94%)	60 (72%)	<0.01**
Knows fever postpartum is a danger sign	10 (10%)	3 (3.6%)	0.09
Midwife discussed newborn problems during antenatal care	81 (82.7%)	19 (23.5%)	<0. 01**
Plans to use family planning	58 (63.0%)	24 (31.6%)	<0. 01**

*p ≤ .05; **p ≤ .01

### Medical record review

A review of medical records was also conducted to examine health outcomes of the mother and newborn. The majority of births occurred at the hospital with no significant difference between groups of women giving birth at home. Women who reported delivering at home were interviewed and their antenatal cards reviewed at the time of their postnatal check. Only 4% of women enrolled in group ANC (*n* = 4) and 3.6% of women enrolled in individual care (*n* = 3) delivered at home (*p* = 0.89). One stillbirth occurred in each group. All women received adequate coverage of tetanus toxoid as well as intermittent preventive therapy for malaria during their pregnancy. There were no significant differences in Apgar scores at one and 5 minutes or in birth weight between groups (Table 7).

**Table 7:** Maternal & Newborn Outcomes

Newborn Outcomes	Group (*n* = 100)	Individual (*n* = 83)	*p*-value
Apgar at 1 min Mean (SD)	7.7 (1.5)	7.6 (1.2)	0.65
Apgar at 5 min Mean (SD)	9.2 (1.8)	9.0 (1.2)	0.52
Birth weight in grams Mean (SD)	3103 (491.1)	3073 (487.9)	0.68
Low birth weight (<2500 g)	6 (6%)	6 (7.2%)	0.74
Respiratory distress	4 (4%)	4 (4.9%)	0.8
Sepsis	1 (1%)	1 (1.3%)	0.86
Maternal Outcomes			
Number of ANC visits Mean (SD)	7.4 (1.3)	6.4 (2.0)	<0. 01**
Caesarean section	17 (17%)	12 (14.5%)	0.64
Hgb immediately before delivery Mean (SD)	11.5 (1.2)	11.6 (1.1)	0.91
Pre-eclampsia	2 (2%)	1 (1.2%)	0.67
Postpartum hemorrhage	1 (1%)	2 (2.4%)	0.46

*p ≤ .05; **p ≤ .01

## Discussion

Study results indicate the efficacy of group ANC to provide important information in a manner which improves women’s ability to retain, understand, and utilize health messages.

Birth preparedness and complication readiness is a strategy widely used in low-resource countries to promote the timely use of skilled maternal and neonatal care, especially during childbirth. The cornerstones of birth preparedness and complication readiness include such behaviors as raising awareness of danger signs; improving problem recognition and reducing delay in deciding to seek care; choosing a birth location and provider in advance; knowing the location of the nearest skilled provider; obtaining basic safe birth supplies; and identifying someone to accompany them to the facility when labor begins [39]. Women in our study enrolled in group care reported significantly higher rates for discussing with the midwife where to deliver and arranging emergency transport in case of a problem as well as to have saved money for their birth.

Exclusive breastfeeding up to 6 months of age is recommended by WHO for all newborns [40]. Women enrolled in the group ANC model of care were able to state significantly more positive practices associated with exclusive breastfeeding than women in individual care.

Only a minority of women (23.5%)enrolled in individual care reported discussing newborn problems with the midwife during ANC visits. Yet the majority of maternal and newborn deaths in the world occur in the first month after birth with half of all maternal deaths occurring in the first 24 h [41] and 66% in the first week following birth [42].

Additionally, our findings reflect the high level of care already being delivered at this facility. Both groups exhibited high literacy related to not giving supplemental feedings or water to their newborn. There was also no significant difference between women enrolled in group or individual ANC who reported they were instructed to sleep under mosquito netting and take iron tables to prevent anemia. Overall, women in both groups were able to identify the majority of danger signs to watch for during pregnancy.

While the number of miscarriages (6 women in individual ANC vs. 4 women in group ANC) and transfer of care to another facility (7 women in each group) is similar between groups, 20% of women in individual ANC (*n* = 24) vs. 7% of women in the group ANC (*n* = 9) failed to return for care after their first or second appointment with the midwife. Unfortunately, we were not able to follow up with these women to understand the reasons for this higher dropout rate from women enrolled in individual care.

### Limitations

There are several limitations to this research. The women recruited for this study represented a convenience sample from one district hospital in one urban region of Ghana, minimizing the ability to generalize our findings. The same midwives provided care to women in group ANC and individual ANC in this study; therefore, it is possible some of the techniques learned during the training of trainers related to communication and information sharing were adopted and used by the midwives during individual antenatal care. The research assistant was not blinded to the type of ANC women were receiving potentially biasing her during data collection.

Despite these limitations, our findings provide evidence of the potential impact of providing ANC to women using a group care model in a low-resource global setting. Our findings support the hypothesis that group ANC can effectively improve women’s comprehension of educational messages received during pregnancy.

While there is a body of evidence developing on how group care impacts patient satisfaction and improves communication between providers and pregnant women [14, 16, 25], exploration into what components of group care make it more effective in changing health behaviors is lacking in the literature. Future research is needed to understand why women drop out of care and whether group ANC is more effective at keeping women engaged in maternal health services long term. Future experimental design research is needed to examine the impact of group ANC on maternal and newborn outcomes and should expand into both peri-urban and rural areas to examine the effectiveness of group ANC on diverse populations.

## Conclusion

Group ANC offers a high impact alternative to standard ANC with the potential to improve quality, generate demand for services, increase healthy behaviors and promote respectful care in low-resource settings.

Patient-provider interactions during ANC provide the opportunity to identify and treat numerous problems as well as a setting to improve women’s health literacy on how to prevent and recognize problems in themselves, prepare for birth, and care for their soon-to-be-born baby. Findings from our study add to a growing body of literature on the use of group ANC to improve maternal and newborn care.

## Additional files

Additional file 1: Chart Review Guide. (PDF 148 kb)

Additional file 2: Demographic Data. (PDF 88 kb)

Additional file 3: Summative Evaluation for women. (PDF 263 kb)

## Abbreviations

ANC: antenatal care
BEmONC: Basic emergency obstetric care
USAID: United States agency for international development
WHO: World Health Organization
