# Supplementary material for: Improving health literacy through group antenatal care: a prospective cohort study
Source: BMC Pregnancy Childbirth. 2017 Jul 14;17:228. doi: 10.1186/s12884-017-1414-5 (PMC5513199; doi:10.1186/s12884-017-1414-5)
Supplement: Supplementary file 1 — Chart Review Guide. (PDF 148 kb) [file 12884_2017_1414_MOESM1_ESM.pdf]

NEW AVENUES TO INCREASE THE USE OF SKILLED BIRTH ATTENDANTS IN GHANA – AIM 3

CHART REVIEW GUIDE

This form will be used to collect data after the woman has delivered. Data will be retrieved from her medical record (if a facility birth has occurred), or from an interview with the woman if she delivered at home.

Date of Chart Review:____

Date of Interview:____

Date of Delivery:____

Where did the woman deliver:____

Participant ID Number:______________________

Live Infant: Yes/No

Multiple: Yes/No

If no: Stillbirth: Yes/No

Early Neonatal death in the first 7 days: Yes/No

Late Neonatal death in the first 30 days: Yes/No

Apgars: 1 minute__________ 5 minutes_____________________

Birth Weight:____

Mother's Hemoglobin before delivery_______________________

Mother's Hemoglobin after delivery_________________________

Type of Delivery:

- ☐ Vaginal
- ☐ Forceps/Vacuum
- ☐ Caesarean Section

Mother Alive: Yes/No

1. **Did any of the following occur:**
   a. **Maternal morbidities:**

      i. Pre-eclampsia/eclampsia Yes/No

      ii. Post partum haemorrhage Yes/No

      iii. Anemia Yes/No

      iv. Ruptured uterus Yes/No

      v. Sepsis Yes/No

   b. **Neonatal morbidities:**

      i. Respiratory distress Yes/No

      ii. Sepsis Yes/No
