# Supplementary material for: Improving health literacy through group antenatal care: a prospective cohort study
Source: BMC Pregnancy Childbirth. 2017 Jul 14;17:228. doi: 10.1186/s12884-017-1414-5 (PMC5513199; doi:10.1186/s12884-017-1414-5)
Supplement: Supplementary file 2 — Demographic Data. (PDF 88 kb) [file 12884_2017_1414_MOESM2_ESM.pdf]

Date:_____________________________ Participant ID Number:_______________

DEMOGRAPHICS

These data will be collected from women in both the intervention group and the control group when they are enrolled in the study.

1. How old were you on your last birthday? ___________________
2. What is your religion?
   - ☐ Christian
   - ☐ Muslim
   - ☐ Buddhist
   - ☐ Pagan
   - ☐ None
3. What is your occupation?________________________________
4. What is your marital status?
   - ☐ Married
   - ☐ Single
   - ☐ Divorced
   - ☐ Widowed
5. Have you ever attended school?
   - ☐ Yes
   - ☐ No
6. If yes, what is the highest level/grade of school you attended?_________________
7. Do you know how to read?
   - ☐ Yes
   - ☐ No
8. What language do you speak (check all that apply):
   - ☐ Twi
   - ☐ English
   - ☐ Housa
   - ☐ Other____
9. Do you know how to write?
   - ☐ Yes
   - ☐ No
10. How many times have you been pregnant?____________________
11. How many living children do you have?_______________________________
12. Where was each of your children born?

Child 1 (oldest)	☐ Home	☐ Hospital
Child 2	☐ Home	☐ Hospital
Child 3	☐ Home	☐ Hospital
Child 4 (youngest)	☐ Home	☐ Hospital

(Add more below as needed)

____

____

13. How far away from Manhyia District Hospital do you live? (how long does it take you to travel to Manhyia for care (in minutes)and how do you get here? (type of transport i.e) car, walk, taxi, tro-tro)

____

____
