# Supplementary material for: Improving health literacy through group antenatal care: a prospective cohort study
Source: BMC Pregnancy Childbirth. 2017 Jul 14;17:228. doi: 10.1186/s12884-017-1414-5 (PMC5513199; doi:10.1186/s12884-017-1414-5)
Supplement: Supplementary file 3 — Summative Evaluation for women. (PDF 263 kb) [file 12884_2017_1414_MOESM3_ESM.pdf]

NEW AVENUES TO INCREASE THE USE OF SKILLED BIRTH ATTENDANTS IN GHANA – AIM 3

SUMMATIVE EVALUATION

This form will be used to collect data after the woman has delivered. Data will be retrieved from her medical record (if a facility birth has occurred), her antenatal card, and from a structured survey either in-person at a post-partum appointment or via telephone survey. **These data will be collected from women in both the intervention group (group care) and the control group (individual care).**

Date: _______________

Participant ID Number:_______________

This is the final questionnaire in the study you have been a part of during your pregnancy. Now that your baby is born we would like to obtain some information from your antenatal care book and ask you a few questions. This interview is completely voluntary and you may refuse to answer any or all of the questions and you may stop the interview at any time. Whether you participate in this interview or not will not affect the health care services you receive at any future point in time.

Information from this interview will be provided to researchers for analyses, but neither your name nor the date of services will be on any shared information, so your identity will remain completely confidential.

1.) Delivery Date__________

2.) Do you have an antenatal care card/book or a vaccination card with you today?

If **YES**, ask to see the card/book.

CHECK in the card/book.

3.) Indicate whether there is any note or record of the woman having received tetanus toxoid.

- ☐ Yes, 1 time
- ☐ Yes, 2 times
- ☐ Yes, 3 or more times
- ☐ No record

4.) Indicate whether there is any note or record of the woman having received ITP?

☐ Yes, 1 time

☐ Yes, 2 times

☐ Yes, 3 times

☐ Yes, 4 times

☐ No record

5.) Was this your first experience receiving ANC?

☐ Yes

☐ No

6.) If no, tell me about your previous experience with ANC?

Probes: Where did you receive ANC?

Probes: How did you find it?

7.) Now that you have had this time with the midwife for ANC, how did you find it?

____
____
____
____

Probes: Can you speak about it?

____
____
____
____

Probes: Can you give some specific examples?

____
____
____
____

CHOOSE TO ASK #8 OR #9 DEPENDING ON WHICH ARM OF THE STUDY THE WOMAN PARTICIPATED IN (GROUP VS. INDIVIDUAL CARE)

FOR WOMEN RECEIVING INDIVIDUAL CARE:

8.) You received ANC in the traditional way by seeing the midwife individually in the exam room. The other group of women was assigned to group care where they received ANC and teaching in a group of 10-12 women of the same gestational age. This group spent about 1 hour with the midwife at each visit. Which of these would you prefer?

Probe: Can you tell me a little more about that? Why?

FOR WOMEN RECEIVING GROUP CARE:

9.) You received ANC in a group format. The other group of women was assigned to individual care where they received ANC and teaching privately from the midwife. This group spent about 5-10 minutes with the midwife at each visit. Which of these would you prefer?

Probe: Can you tell me a little more about that? Why?

10.) Did you have any concerns about your privacy during your antenatal care?

1	2
**Yes**	**No**

Probe: If yes, can you tell me about your concerns?

11.) How satisfied were you with the courtesy and consideration shown to you by the midwife during your antenatal care?

1	2	3
**Satisfied**	**Undecided**	**Dissatisfied**

12.) How satisfied were you with the skills and competencies of the midwife during your antenatal care?

1	2	3
**Satisfied**	**Undecided**	**Dissatisfied**

12.) How satisfied were you with the midwife's willingness to listen to your explanation of your problems?

1	2	3
**Satisfied**	**Undecided**	**Dissatisfied**

13.) How satisfied were you with the advice given to you by the midwife?

1	2	3
**Satisfied**	**Undecided**	**Dissatisfied**

14.). How satisfied were you with the waiting time after your arrival at the health center?

1	2	3
**Satisfied**	**Undecided**	**Dissatisfied**

15.) How satisfied were you with the length of consultation time with the midwife?

1	2	3
**Satisfied**	**Undecided**	**Dissatisfied**

16.) The midwife was respectful of me during my antenatal visits?

1	2	3
**Satisfied**	**Undecided**	**Dissatisfied**

17.) The midwife was polite to me during my antenatal visits?

1	2	3
**Agree**	**Neither Agree or Disagree**	**Disagree**

18.) Overall, which of the following best describes your opinion of the services you received during antenatal care:

1	2	3
**Very satisfied**	**More or less satisfied**	**Not satisfied**

KNOWLEDGE GAINED

19.) Did you receive iron pills, folic acid, or a prescription for iron/folic acid during your pregnancy?

☐ Yes

☐ No

☐ Don't know

20.) Did the midwife explain to you how to take the iron or folic acid pills?

☐ Yes

☐ No

☐ Don't know

21.) Did the midwife discuss with you the side effects of iron/folic acid pills?

☐ Yes

☐ No

☐ Don't know

22.) Please tell me any side effects of the iron pill you know of?

☐ Nausea

☐ Constipation

☐ Black Stools

☐ Other____

23.) During your antenatal care, did the midwife recommend you sleep under a mosquito net?

☐ Yes

☐ No

☐ Don't know

24.) During your antenatal care, did the midwife talk to you about nutrition or what is good for you to be eating during your pregnancy?

☐ Yes

☐ No

☐ Don't know

25.) During your antenatal care, did the midwife talk with you about any signs that should warn you about problems or complications during pregnancy?

☐ Yes

☐ No

☐ Don't know

26.) What are some things you learned in ANC that helped you to prevent problems during your pregnancy?

☐ Rest often

☐ Sleep under a mosquito net

☐ Eat an extra meal every day

☐ Drink plenty of fluids

☐ Take iron and folic acid tablets

☐ Go to ANC visits

☐ Watch for problems and report to clinic with problems

☐ Practice safe sex

☐ Plan next pregnancy

☐ Other____

☐ Don't know any

27.) Please tell me any signs of complications (danger signs) that you know of. Check all responses the client mentions. You may probe with "anything else"?

☐ Vaginal Bleeding

☐ Fever

☐ Pain in breasts or abdomen

☐ Swollen face or hands

☐ Headache or blurred vision

☐ Reduced or no fetal movement

☐ Other____

☐ Don't know any

28.) What did the midwife advise you to do if you experienced any of the signs of complications?____

____

____
____
____

29.) Did the midwife discuss things you should have in preparation for this delivery?

☐ Yes

☐ No

☐ Don’t Know

30.) If yes, tell me some of the things you did to prepare for the delivery?

☐ Arranged for emergency transport if needed

☐ Money

☐ Layette for baby

☐ Cleaned area in the house for newborn

☐ Obtained supplies for the birth

☐ Kept self clean (bathing)

☐ Eating and drinking light foods

☐ Watched for problems

31.) Did you have money set aside for the delivery?

☐ Yes

☐ No

32.) If yes, do you think you had enough set aside?

☐ Yes

☐ No

☐ Don’t know

33.) Did you talk to your midwife about where you planned to delivery your baby?

☐ Yes

☐ No

34.) What are some things you can do **for yourself** to prevent problems after your baby is born?

☐ Bathe every day

☐ Pass urine often

☐ Wipe from front to back

☐ Eat at least 4x day

☐ Drink plenty of liquids

☐ Rest

☐ Sleep under an insecticide treated bednet

☐ Watched for problems

☐ Go for a postpartum check up

35.) Do you know any complications during or immediately following childbirth?

☐ Yes

☐ No

36.) If yes, what danger signs do you know?

☐ Excessive bleeding ☐ Fever

☐ Other____

37.) Did the midwife give you advice on the importance of exclusive breastfeeding?

☐ Yes

☐ No

☐ Don't know

38.) How many months did the midwife recommend that you exclusively breastfeed, that is, that you do not give your baby any fluids or food in addition to breast milk?

☐ Between 4-6 months

☐ Six months

☐ Seven months

☐ Other____

☐ Don't know

39.) During antenatal care did the midwife talk with you about using family planning after the birth of your baby?

☐ Yes

☐ No

☐ Don’t know

40.) Do you plan to use family planning?

☐ Yes

☐ No

☐ Don’t know

41.) What method of family planning will you use or are you using now?

☐ LAM

☐ Pills

☐ Depo-provera

☐ IUD

☐ Abstinence

☐ Implanon

☐ Condoms

☐ Withdrawal

☐ Don’t know

☐ None

☐ Other____

42.) Tell me what you know about the Lactation Amenorrhea Method (LAM).

☐ Start to breastfeed as soon as possible after birth

☐ Breastfeed every 2-4 hours during the day

☐ Breastfeed at least once during the night

☐ Give breastmilk until baby is 6 months old

☐ Do not give bottle

☐ Do not give water

☐ Do not give supplemental feeds

☐ Nothing

43.) How soon do you plan to get pregnant or have another baby?

____

44.) Have you had a post-partum check-up at the clinic?

☐ Yes

☐ No

If no, do you have an appointment scheduled for a post-partum check-up?

☐ Yes

☐ No

45.) During antenatal care did the midwife talk with you about newborn problems you should watch for?

☐ Yes

☐ No

☐ Don't know

46.) What problems in your newborn did she tell you to watch for?

☐ Poor or no sucking

☐ Infant has trouble breathing

☐ Fits or convulsions

☐ Fever and pus in eyes

☐ No crying

☐ Fever and pus in cord stump

47.) Where did you deliver your last baby?

☐ Home

☐ Manhyia District Hospital

☐ Other Hospital/Clinic/Maternity Home (name)________________________________

48.) Who attended your last delivery?

☐ Certified Midwife

☐ Nurse

☐ Physician

☐ Traditional Birth Attendant

☐ Family Member

☐ No one, I was alone

☐ Other____

49.) Where did you PLAN to deliver this baby?

☐ Home

☐ Manhyia District Hospital

☐ Other Hospital/Clinic/Maternity Home (name)________________________________

50.) Where DID you deliver this baby?

☐ Home

☐ Manhyia District Hospital

☐ Other Hospital/Clinic/Maternity Home (name)________________________________

51.) Did the group or individual care influence you on where to deliver?_______________

If yes, how:

____

____

____

52.) Did you experience any problems with your labor and/or delivery?

☐ Yes

☐ No

☐ Don’t know

53.) If yes, what problems did you experience?

☐ Antenatal bleeding

☐ Postpartum haemorrhage

☐ Obstructed labor

☐ Ruptured uterus

☐ Pre-eclampsia

☐ Eclampsia

☐ Sepsis

☐ Caesarean section

54.) Did your newborn experience any problems?

☐ Stillborn

☐ Neonatal death

☐ Sepsis

☐ Seizures

☐ Tetanus

55.) Is there anything else you would like to tell us about your experience with antenatal care?____

____

____

____

____

____
